# Mesenchymal stromal cell-derived small extracellular vesicles promote neurological recovery and brain remodeling after distal middle cerebral artery occlusion in aged rats

**DOI:** 10.1007/s11357-021-00483-2

**Published:** 2021-11-10

**Authors:** Danut-Adrian Dumbrava, Roxana Surugiu, Verena Börger, Mihai Ruscu, Tobias Tertel, Bernd Giebel, Dirk M. Hermann, Aurel Popa-Wagner

**Affiliations:** 1 Department of Neurology, University Hospital Essen, University of Duisburg-Essen, Essen, Germany; 2 Experimental Research Center in Normal and Pathological Aging (ARES), University of Medicine and Pharmacy, Craiova, Romania; 3 Institute for Transfusion Medicine, Essen, Germany; 4 Griffith University Menzies Health Institute of Queensland, Gold Coast Campus, Southport, QLD 4222 Australia

**Keywords:** Aging, Angiogenesis, Exosome, Ischemic stroke, Macrophage, Neurogenesis, Stem/precursor cell, Permanent focal cerebral ischemia

## Abstract

Small extracellular vesicles (sEVs) obtained from mesenchymal stromal cells (MSCs) promote neurological recovery after middle cerebral artery occlusion (MCAO) in young rodents. Ischemic stroke mainly affects aged humans. MSC-sEV effects on stroke recovery in aged rodents had not been assessed. In a head-to-head comparison, we exposed young (4–5 months) and aged (19–20 months) male Sprague–Dawley rats to permanent distal MCAO. At 24 h, 3 and 7 days post-stroke, vehicle or MSC-sEVs (2 × 10^6^ or 2 × 10^7^ MSC equivalents/kg) were intravenously administered. Neurological deficits, ischemic injury, brain inflammatory responses, post-ischemic angiogenesis, and endogenous neurogenesis were evaluated over 28 days. Post-MCAO, aged vehicle-treated rats exhibited more severe motor-coordination deficits evaluated by rotating pole and cylinder tests and larger brain infarcts than young vehicle-treated rats. Although infarct volume was not influenced by MSC-sEVs, sEVs at both doses effectively reduced motor-coordination deficits in young and aged rats. Brain macrophage infiltrates in periinfarct tissue, which were evaluated as marker of a recovery-aversive inflammatory environment, were significantly stronger in aged than young vehicle-treated rats. sEVs reduced brain macrophage infiltrates in aged, but not young rats. The tolerogenic shift in immune balance paved the way for structural brain tissue remodeling. Hence, sEVs at both doses increased periinfarct angiogenesis evaluated by CD31/BrdU immunohistochemistry in young and aged rats, and low-dose sEVs increased neurogenesis in the subventricular zone examined by DCX/BrdU immunohistochemistry. Our study provides robust evidence that MSC-sEVs promote functional neurological recovery and brain tissue remodeling in aged rats post-stroke. This study encourages further proof-of-concept studies in clinic-relevant stroke settings.

**Supplementary Information:**

The online version contains supplementary material available at 10.1007/s11357-021-00483-2.

## Introduction

Small extracellular vesicles (sEVs) obtained from mesenchymal stromal cells (MSCs) hold great promise as restorative stroke treatments. Following early reports of enhanced neuronal plasticity and neurological recovery following delivery of MSC-derived sEVs in rats exposed to middle cerebral artery occlusion (MCAO) [1, 2], our group previously showed that MSC-sEVs very similarly effectively increased motor-coordination recovery, long-term neuronal survival, periinfarct angiogenesis, and neurogenesis as their parental MSCs when administered starting 24 h after MCAO in mice [3, 4].

sEVs, which comprise exosomes (70–150 nm), play important roles in intercellular communication in physiological and pathophysiological processes [5]. sEVs carry complex signal cargos and efficiently modify disease processes when obtained from the right cell type raised under appropriate culturing conditions [6, 7]. sEV-based therapies have various advantages over cell therapies [8]: sEVs are not self-replicating and lack endogenous malignant transformation risks. Due to their small size, sEV products can be sterilized by filtration, and their handling is much easier than that of cellular therapeutics. sEVs can hardly sense environmental conditions, and their biological activity can be predicted more precisely than that of cells.

In view of their unique potential, MSC-sEVs are rapidly approaching clinical trials. In the absence of any therapeutic alternatives, we have previously treated for the first time worldwide a steroid-refractory acute graft-versus-host disease patient with escalating MSC-sEV doses [9]. In the lack of side effects, a two-week MSC-sEV treatment reduced graft-versus-host disease (GvHD) symptoms for more than 4 months. Ischemic stroke mainly affects aged humans. Importantly, the effects of MSC-sEVs have so far been evaluated in young, mostly otherwise healthy rats and mice [1, 2, 4, 10–18]. To date, MSC-sEV effects on stroke recovery have not yet been assessed in aged rodents. In a head-to-head comparison, we here exposed young and aged rats to permanent distal MCAO, which results in purely cortical brain infarcts, and intravenously administered MSC-sEVs to these rats starting 24 h post-MCAO. Neurological recovery was evaluated by rotating pole and cylinder tests, and brain remodeling was studied by immunohistochemistry and BrdU incorporation analysis. Our study provides first evidence that MSC-sEVs promote post-ischemic neurological recovery and brain remodeling in aged rats.

## Experimental procedures

### Ethics and data availability

Experiments were performed with local approval (Institutional Animal Care and Use Committee of the University of Medicine and Pharmacy Craiova (#112–15-11–2017) in accordance to E.U. guidelines (Directive 2010/63/EU) for the care and use of laboratory animals and local institutional guidelines. In adherence to ARRIVE guidelines, experiments were strictly randomized. Examiners performing data analyses, including behavioral tests, were fully blinded for experimental conditions across the study. The data that support the findings of this study are available from the last author upon reasonable request.

### Statistical planning

Statistical planning was done by a sample size calculator (https://www.sphanalytics.com/sample-size-calculator-using-average-values/). Assuming an alpha error of 5% and a beta error (1, statistical power) of 20%, sample size calculation determined that 15 animals were needed per group for behavioral and histochemical analyses, provided that sEVs modified the mean value by 20% and that the standard deviation of the data sample was 20% of the mean value (effect size: 1). The mortality rate in this study was higher for aged rats (22%) than young rats (13%). For this reason, the group size of 15 rats per group was enlarged to 18 for aged rats to ensure comparable group sizes across this study.

### Animals

A total of 99 young (4 to 5 months) and aged (19 to 20 months) male Sprague–Dawley rats, bred in the animal facility of the University of Medicine and Pharmacy Craiova, were included. Body weights ranged from 310 to 400 g for young rats and from 550 to 700 g for aged rats. Rats were kept in a regular 12:12 h light/dark rhythm (light period from 07.00 to 19.00 h) with free access to food and water at an ambient temperature of 22 °C (40–60% humidity).

### Expansion and characterization of MSCs

Human MSCs were raised with informed consent from bone marrow samples of a healthy donor (source 41.5), as previously reported [16]. T

he bone marrow sample was obtained via our university hospital internal bone marrow transplantation unit in conjunction of the Westdeutsche Spender Zentrale (https://www.wsze.de/startseite/index.php). According to their policies, as agreed by the local Ethics Commission and provided the donor’s agreement in an informed consent, small bone marrow aliquots can be used for research purposes. The aliquots are anonymized. Hence, donors are not specifically enrolled for our research purposes. No details about the donor’s sex or age were provided to us. MSCs were expanded in low glucose Dulbecco’s modified Eagle medium (DMEM) (Lonza) supplemented with 10% human platelet lysate (hPL; in-house produced), 100 U/ml penicillin–streptomycin-L-glutamine (Thermo Fisher Scientific, Waltham, MA, USA), and 5 IU/ml heparin (Heparin-Natrium-25000, Ratiopharm, Ulm, Germany) and passaged at approximately 80% confluency. In passage three, MSCs were characterized according to International Society of Cell and Gene Therapy (ISCT) standards, as described before [19]. The presence of MSC markers on these cells and their osteogenic and adipogenic differentiation potential have previously been demonstrated [16]. Starting at passage 3, conditioned media were harvested every 48 h from MSCs cultured under normoxic conditions (21% O_2_), as previously described [9]. The conditioned media were centrifuged at 2,000 g for 15 min to remove cell debris and stored at − 20 °C until usage. Only conditioned media tested negative for mycoplasma contamination were used. Finally, the MSC-EVs were adjusted that 1 mL final sample contained the sEV yield prepared from conditioned media of approximately 4 × 10^7^ MSC equivalents and defined as 1 unit. The corresponding particle and protein concentration are given in Supplementary Table 1.

### Preparation of MSC-sEVs

Conditioned media were simultaneously thawed and processed. To remove debris and larger vesicles, supernatants were centrifuged at 6,800 g for 45 min in an Avanti centrifuge (JS-5.3 rotor; k-factor: 7,730; Beckman-Coulter). Next, sEVs were concentrated by polyethylene glycol 6000 (PEG) precipitation exactly as described before [9]. Finally, sEVs were washed in 0.9% NaCl and re-precipitated by ultracentrifugation at 110,000 g for 130 min (Ti45 rotor, k-factor: 133). MSC-sEV samples were dissolved in 10 mM Hepes/0.9% NaCl (Thermo Fisher Scientific) (4 × 10^7^ cell equivalents per mL, which were defined as 1 U) and stored at − 80 °C. Due to the large amounts of sEVs needed as a consequence of the animals’ body weights, two independent sEV preparations were performed (preparations A and B), which were administered to young and aged rats, respectively.

### Characterization of MSC-sEVs by nanoparticle tracking analysis and imaging flow cytometry

MSC-sEV preparations were characterized according to current ISEV guidelines [20]. Particle concentration and size were measured by nanoparticle tracking analysis (NTA; Particle Metrix, Meerbusch, Germany), as described previously [21]. The protein concentration was determined by a standardized bicinchoninic acid (BCA) assay (Pierce, Rockford, IL, USA). The particle concentration, size, protein concentration, and purity are shown in Suppl. Table 1. Using Western blots, we had previously shown the presence of the exosome markers CD9, CD63, CD81, and syntenin and the absence of the cytosolic markers calnexin and prohibitin in sEV samples [16]. By transmission electron microscopy, we provided evidence that sEVs within preparations had the typical appearance and size of exosomes [16]. By imaging flow cytometry using the AMNIS ImageStreamX Mark II Flow Cytometer (Luminex, Seattle, WA, USA), we now show the presence of CD9^+^, CD63^+^, and CD81^+^ vesicles in sEV samples (Suppl. Table 2). The gating strategy used for the sEV analyses is presented in Suppl. Figure 1. Antibodies used for flow cytometry of sEVs are listed in Suppl. Table 3.

**Fig. 1:**
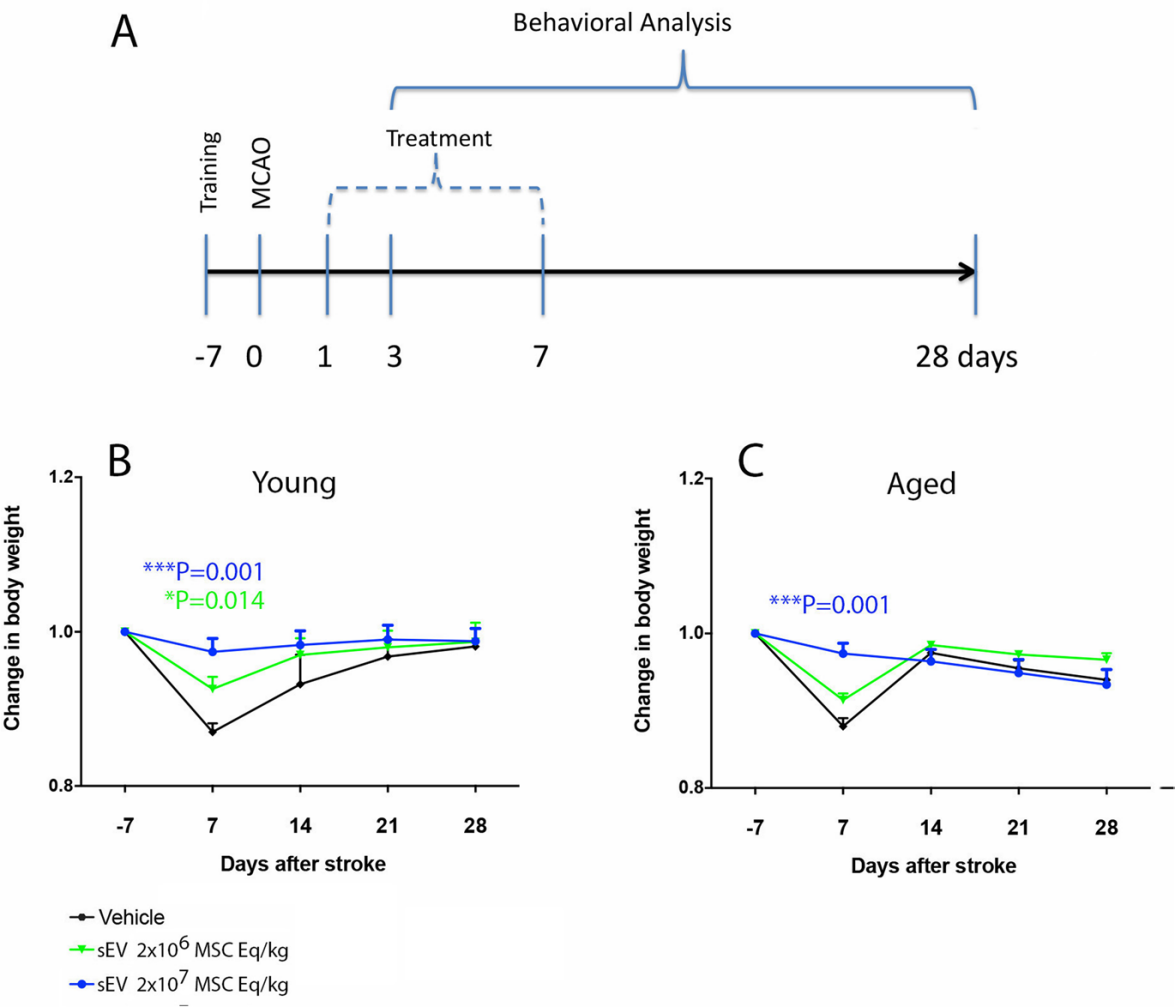
Experimental design and time course of body weight changes in young and aged rats exposed to permanent distal middle cerebral artery occlusion (MCAO). **A** Experimental design showing the time-line of experimental interventions. Rats (*n* = 15 animals/group) received intravenous injections of mesenchymal stromal cell (MSC)-derived small extracellular vesicles (sEVs) at 1, 3, and 7 days post-MCAO. Motor-coordination deficits were evaluated by behavioral analysis from 3 to 28 days post-MCAO (i.e., from 2 to 27 days post-treatment onset). Rats were sacrificed after 28 days for brain tissue analysis. **B**,** C** Time course of body weight changes in young and aged rats exposed to permanent distal MCAO. Body weight decreased in young and aged vehicle-treated rats in the first 7 days post-stroke. Body weight fully recovered within 14–21 days in all groups. Note that MSC-sEVs prevented the weight loss at 7 days in young and aged rats. Data are means ± SEM values

### Behavioral testing

The animal experiments involved two persons, one person who did the surgery and was in charge of animal handling and another one who performed the behavioral tests. Behavioral testing was performed from 9 to 11 a.m. A baseline examination was performed 1 week prior to MCAO. Behavioral tests were repeated at 3 days post stroke (that is, 2 days post-MSC-sEV delivery), 7 days, 14 days, 21 days, and 28 days post-stroke.

#### Rotating pole test

The rotating pole task assesses coordination and sensorimotor function. Each rat was tested for its ability to cross a rotating (3 or 6 rpm) horizontal rod. Test performance was scored as previously described by our group [22]. Briefly, the time needed for each rat to traverse the rotating pole and join a group of rats visible at the finish line was measured at both speeds.

#### Cylinder test

The cylinder test examines the asymmetry of sensorimotor performance of both forelimbs. Each rat was videotaped for 5 min while placed in a 20 cm diameter and 40 cm height glass cylinder, in which vertical exploration of the walls was measured by counting the number of wall contacts of each forelimb, as previously described by us [23]. The asymmetry index was calculated as (left − right)/(left + right) ratio, where left and right are the number of wall contacts of the left (lesion contralateral) and right (lesion ipsilateral) forelimbs, respectively.

### Animal surgery

Prior to surgery, rats were fasted overnight to reduce the blood glucose levels. After craniotomy, the right middle cerebral artery (MCA) was exposed, slowly lifted with a tungsten hook (Fine Science Tools, Heidelberg, Germany) attached to a micromanipulator (Märzhäuser Precision Micro-manipulator Systems, Applied Scientific Instrumentation, Eugene, OR, USA), and thermocoagulated. Both common carotid arteries were then occluded by tightening pre-positioned thread loops for 90 min. Throughout surgery, anesthesia was maintained by spontaneous inhalation of 1–1.5% isoflurane in a mixture of 75% nitrous oxide and 25% oxygen. Body temperature was controlled at 37 °C by a homeothermic blanket system (Harvard Apparatus; Cambridge, MA, USA). Local changes in blood flow were monitored using a laser Doppler flow device (Perimed, Stockholm, Sweden). A decrease in laser Doppler flow signals to < 20% of control values was considered as successful MCAO. After 90 min, the common carotid arteries were re-opened. Soft tissue wounds and skin were carefully closed using 5–0 nylon suture. For pain relief, buprenorphine (0.3 mg/kg) was s.c. administered twice at a 6-h interval for 3 days post-stroke. Moistened food was provided for the first 3 days post-surgery. At 1 days, 3 days and 7 days post-stroke, vehicle, or sEVs prepared from conditioned media of bone marrow-derived MSCs were administered through the animals’ tail vein at two doses (2 × 10^6^ or 2 × 10^7^ MSC equivalents/kg on each occasion), which were dissolved in 0.9% NaCl. Dose selection was made based on a previous study [4] in which we applied the 100-fold dose (2 × 10^7^ MSC equivalents/kg) we had used before in the patient [9]. To gain further insights into dose–response relationships, we here administered a ten-fold lower dose too (2 × 10^6^ MSC equivalents/kg).

### BrdU labelling

To label newly generated cells, bromodeoxyuridine (BrdU; 50 mg/kg body weight, i.p.; Sigma) was intraperitonally administered daily from day 8 to 18.

### Animal sacrifice

At 28 days post-stroke (27 days post-treatment onset), the rats were deeply anesthetized with 2.5% isoflurane in 75% nitrous oxide and 25% oxygen and perfused with 0.1 M phosphate buffered saline (PBS; pH 7.0) followed by freshly prepared 4% paraformaldehyde in 0.1 M PBS. The brains were removed, post-fixed in 4% paraformaldehyde for 24 h, cryoprotected in 15% glycerol prepared in 10 mmol/l PBS, flash-frozen in isopentane, and stored at − 70 °C until sectioning. 25 µm-thick coronal sections were cut on a freezing microtome. A flow chart of animal experiments is given in Fig. 1A.

### Determination of the infarct volume

To assess the size of the infarct induced by permanent focal cerebral ischemia, brain sections at 500 µm distance (that is, every 20th section) were stained with methyl green/pyronine Y. Images of the stained sections were taken, on which infarct areas were measured using Image J. Infarct areas at various rostrocaudal levels were used for calculating partial infarct volumes. By integrating partial infarct volumes across the brain, total infarct volume was calculated [24, 25]*.*

### Immunohistochemistry

For histochemical analysis of macrophage and microglia accumulation in the periinfarct brain tissue, 25 µm free-floating sections were blocked overnight in 3% donkey serum/ 10 mmol/l PBS/ 0.3% Tween 20 at 4 °C [24, 25]. Sections were immersed for 24 h in monoclonal mouse anti-ED1 antigen (1:300; Abcam, ab31630, Cambridge, UK) and polyclonal rabbit anti-ionized calcium binding adaptor protein (Iba-1) (1:3000; Wako Chemicals, Neuss, Germany) antibodies, followed by incubation in appropriate biotinylated secondary donkey antibodies (Jackson ImmunoResearch Laboratories, West Grove, PA, USA). Sections were stained using the ABC Elite reagents (Vectastain Elite Kit, Vector) in 0.025% 3,3′ diaminobenzidine (DAB) and 0.005% hydrogen peroxide.

For BrdU detection, 25 µm free-floating sections were pre-treated for 2 h with 50% formamide in 0.3 M NaCl containing 10 mM sodium citrate at 65 °C, incubated for 1 h in 2 M HCl at 40 °C, and rinsed in 0.1 M borate buffer (pH 8.5) at room temperature for 10 min. Sections were incubated for 24 h in monoclonal at anti-BrdU (1:1000; BU1/75, ab6326, Abcam), polyclonal guinea-pig anti-doublecortin (1:2000; AB2253, Merck Millipore, Burlington, MA, USA), and monoclonal mouse anti-CD31 (1:1000; clone MEC13.3, 550,274, BD Biosciences, Heidelberg, Germany) at 4 °C, followed by fluorescence detection in goat anti-rat-Cy3, goat anti-guinea-pig Alexa Fluor 488, and goat anti-mouse Alexa Fluor 488 IgG.

### Quantitation of ED1^+^macrophages and Iba1^+^ microglia

A quantitative estimate of the number of ED1^+^ activated macrophages and Iba1^+^ microglia was obtained by cell counting in regions of interest measuring 250 µm × 250 µm, employing a “random-systematic” protocol (random start point for a systematic series of every 10th section through the infarcted volume) using *Image J*. The area occupied by cells of interest was ~ 30% of the total stained infarct area. To eliminate false-positive signals, the cross-sectional area of nuclei was set between 70 and 110 µm^2^ for activated macrophages and 40–70 µm^2^ for Iba1^+^ microglia. The somata of Iba1^+^ microglia measured approximately 5 µm × 18 µm, the surface area of macrophages was ~ 50% larger than that of monocytes. Structures with areas lying outside this range were eliminated from the final count. Hence, monocytes were excluded from cell countings. Cells in the uppermost focal plane were ignored to avoid oversampling errors by counting cell caps [26]. Means were formed for values determined at various rostrocaudal levels of the brain. Data were expressed as cell number per mm^2^.

### Quantitation of CD31^+^/BrdU^+^ blood microvessels

The number of CD31^+^/BrdU^+^ blood microvessels was counted in the periinfarct cortex by evaluating regions of interest measuring 0.7386 mm^2^ in every 10th brain section using a 40 × objective [27]. Means were formed for cell numbers determined at different rostrocaudal levels of the brain. Data were expressed as cell number per mm^2^. Cell counting was done by two independent observers, of which mean values were formed.

### Counting of DCX^+^/BrdU^+^ newborn neurons

DCX^+^/BrdU^+^ newborn neurons were analyzed in the region adjacent to the subventricular zone (SVZ) as previously described [25]. To this end, a sequence of confocal images measuring 161 × 242 µm, which involved the SVZ, was scanned at 0.1 µm steps across the 25 µm-thick sections [24]. DCX^+^/BrdU^+^ double labeled cells were concentrated in these images in an area adjacent to the SVZ that covered ~ 30% of the total image area. Hence, the number of DCX^+^/BrdU^+^ double labeled cells was calculated by multiplying the number of counted cells per image times 3.3. Data were expressed as cell number per mm^2^. Cell counting was done by two independent observers, of which mean values were formed.

### Statistical analysis

For behavioral testing the main effects of time and treatment were evaluated for each age group by 2-way repeated measurement ANOVA followed by Dunett’s multiple comparisons test using GraphPad software. For the analysis of histological data, we used 2-way ANOVA followed Tukey’s multiple comparisons test for assessing the age and treatment effect. Data are mean ± SEM values (longitudinal analyses evaluating body eight changes or behavioral data) or mean ± SD values (cross-sectional analyses evaluating histochemical data). *P* values ≤ 2 × 10^7^ MSC equivalents/kg were considered to indicate statistical significance.

## Results

### MSC-sEV delivery prevents body weight loss post-stroke in young and aged rats

Rats were intravenously treated with vehicle or MSC-sEV (2 × 10^6^ or 2 × 10^7^ MSC equivalents/kg) at 24 h, 3 and 7 days post-MCAO, followed by behavioral analysis of motor-coordination deficits over 28 days, as summarized in the time-line in Fig. 1A. To mitigate problems of food intake in aged animals, animals were fed with moistened, soft food pellets during the first 3 days post-stroke. Nevertheless, aged rats lost ~ 10–15% body weight in the first week following stroke (Fig. 1B, C). MSC-sEVs reduced this weight loss at both doses (2 × 10^6^ or 2 × 10^7^ MSC equivalents/kg) in young rats (Fig. 1B) and at the higher dose (2 × 10^7^ MSC equivalents/kg) in aged rats (Fig. 1C). Body weight returned to baseline within 2–3 weeks post-stroke in all groups (Fig. 1B, C).

### MSC-sEVs promote motor-coordination recovery in young and aged rats

To evaluate the effects of sEVs on functional neurological recovery, rotating pole and cylinder tests were used, which evaluate motor-coordination and forelimb use, respectively (Buchhold et al., 2007; Moldovan et al., 2010). At 24 h post-stroke, all rats exhibited reduced spontaneous motor activity as a consequence of their surgery, which precluded testing of fine motor skills prior to the initiation of sEV treatment. For this reason, behavioral analysis was started at 3 days post-stroke (i.e., 2 days post-treatment onset).

**Fig. 2:**
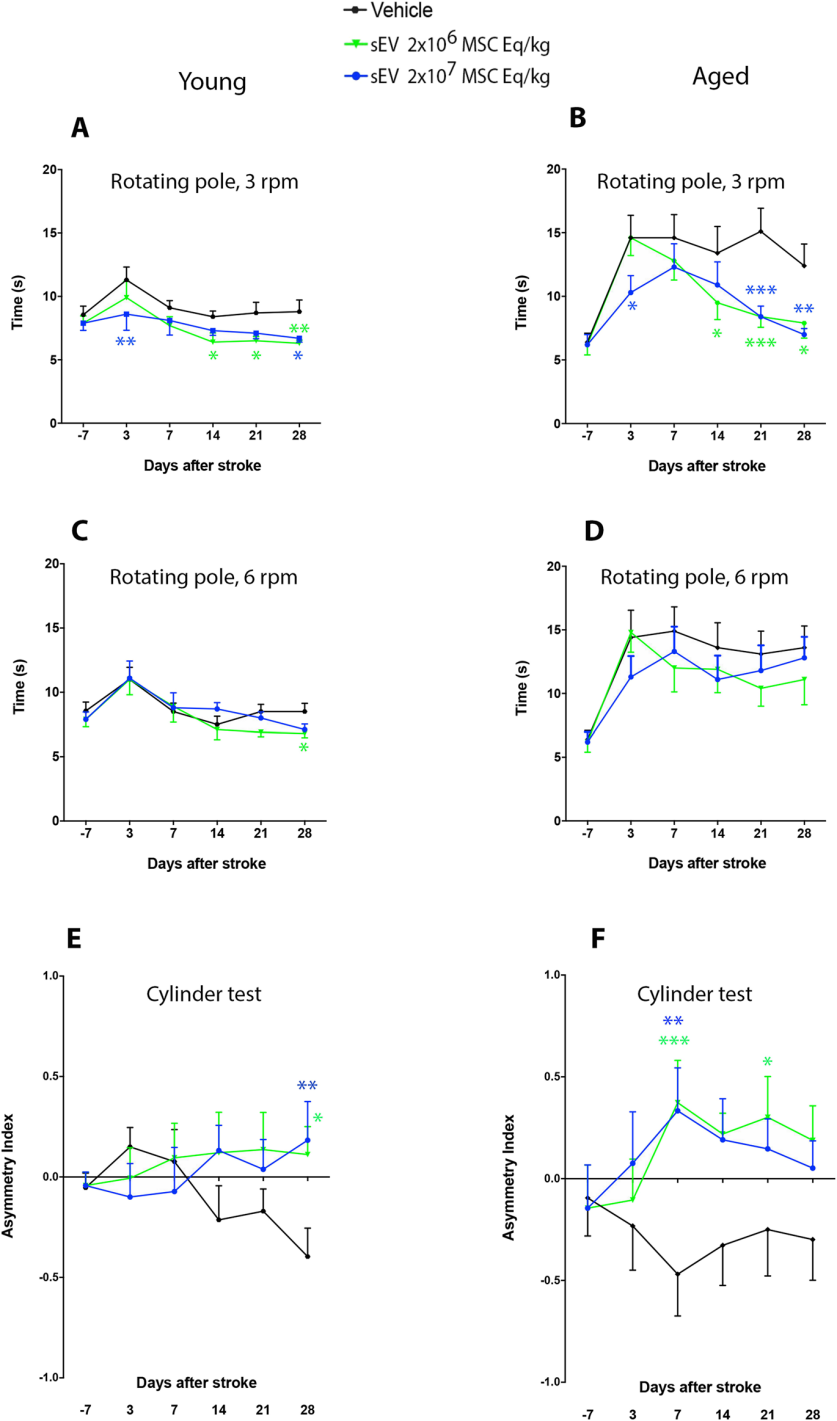
MSC-derived sEVs at both doses promote post-stroke motor-coordination recovery in young and aged rats. Motor-coordination deficits in **A**, **B** the rotating pole test at 3 rpm, **C**, **D** the rotating pole test at 6 rpm, and **E**, **F** the cylinder test of **A**, **C**, **E** young and **B**, **D**, **F** aged rats exposed to permanent distal MCAO which received vehicle or sEVs (2 × 10^6^ or 2 × 10^7^ MSC equivalents/kg) at 1, 3, and 7 days post-stroke (*n* = 15 animals/group). The rotating pole test evaluates the time needed to traverse a pole rotating at a given speed (3 or 6 rpm), and the cylinder test measures the asymmetry of forelimb use, more negative data indicating reduced use of the stroke-affected left limb. Note that the test performance in young and aged rats was robustly improved by low-dose and high-dose MSC-sEVs in **A**, **B** the rotating pole test at 3 rpm and **E**, **F** the cylinder test. In comparison, **C**, **D** the rotating pole test at 6 rpm imposes an elevated task difficulty. This latter test was unable to discriminate behavioral changes between treatment groups in aged rats. Data are mean ± SEM values

*Rotating pole test at 3 rpm.* At 3 days post-stroke (2 days post-treatment onset), the rotating pole test revealed an increased time needed for traversing the rotating pole both in young and aged vehicle-treated rats (Fig. 2A, B). These motor-coordination deficits were more pronounced in aged than young rats (Fig. 2A, B), which is in line with previous studies of our group [22].

In young rats, moderate motor-coordination deficits were noted in rats receiving vehicle at 3 days post-stroke (Fig. 2A). Motor-coordination deficits at 3 days were significantly reduced by high-dose sEVs (Fig. 2A). Motor-coordination deficits spontaneously improved within 7–28 days post-stroke in all young rat groups (Fig. 2A). Compared with rats receiving vehicle, rats receiving low-dose sEVs revealed a significantly enhanced test performance at 14–28 days post-stroke (Fig. 2A). Likewise, rats receiving high-dose sEVs showed a significantly enhanced test performance at 28 days post-stroke (Fig. 2A). In young rats, repeated measurement ANOVA revealed a significant main effect of treatment (*p* = 0.004) and time (*p* = 0.001) on rotating pole test performance at 3 rpm.

Compared with young rats, aged rats exhibited much more robust motor-coordination deficits at 3 days post-stroke (Fig. 2B). Motor-coordination deficits at 3 days were significantly reduced by high-dose sEVs (Fig. 2B). Motor coordination deficits in aged vehicle-treated rats did not reveal major improvements over the recovery period of 28 days, whereas motor-coordination deficits of sEV-treated rats progressively improved starting at 7–14 days post-stroke (Fig. 2B). Compared with rats receiving vehicle, rats receiving low-dose sEVs showed significantly reduced motor-coordination deficits at 14–28 days post-stroke (Fig. 2B). Similarly, rats receiving high-dose sEVs had significantly reduced motor-coordination deficits at 21–28 days post-stroke (Fig. 2B). In view of the more pronounced deficits, the beneficial effects of sEVs at both doses in aged animals exceeded those in young animals. Repeated measurement ANOVA tests revealed significant main effects of treatment (*p* = 0.019) and time (*p* < 0.001) on rotating pole test performance at 3 rpm in aged rats.

#### Rotating pole test at 6 rpm

The rotating pole test exhibits a higher task difficulty at 6 rpm than at 3 rpm. As a consequence of this stronger challenge, young and aged rats revealed more reproducible motor-coordination deficits in the rotating pole test at 6 rpm (Fig. 2C, D). The test performance in the rotating pole test at 6 rpm did not differ between vehicle-treated and sEV-treated rats at 3–21 days post-stroke (Fig. 2C, D). In young rats, low-dose sEVs significantly reduced motor-coordination deficits at 28 days post-stroke (Fig. 2C). Repeated measurement ANOVA revealed a significant main effect of treatment (*p* = 0.040) and time (*p* < 0.001) in young rats.

#### Cylinder test

In the cylinder test, young and aged rats revealed a preference for the non-affected right forelimb after stroke, resulting in a negative forelimb asymmetry index (Fig. 2E,F). This preference developed in a delayed way in young rats (Fig. 2E). It was reproducibly noted at 3 days post-stroke in aged rats (Fig. 2F). Low-dose and high-dose sEVs significantly increased the use of the left paretic forelimb in young and aged rats (Fig. 2E, F). This effect was significant for low-dose and high-dose sEV-treated young rats at 28 days post-stroke (Fig. 2E) and for low-dose and high-dose sEV-treated aged rats at 7 and 28 days (Fig. 2F). Repeated measurement ANOVA revealed a main effect of treatment (*p* = 0.002) and time (*p* = 0.034) on cylinder test performance in young rats and a main effect of treatment (*p* = 0.001) and time (*p* = 0.025) on cylinder test performance in aged rats.

### sEVs do not influence infarct volume

**Fig. 3:**
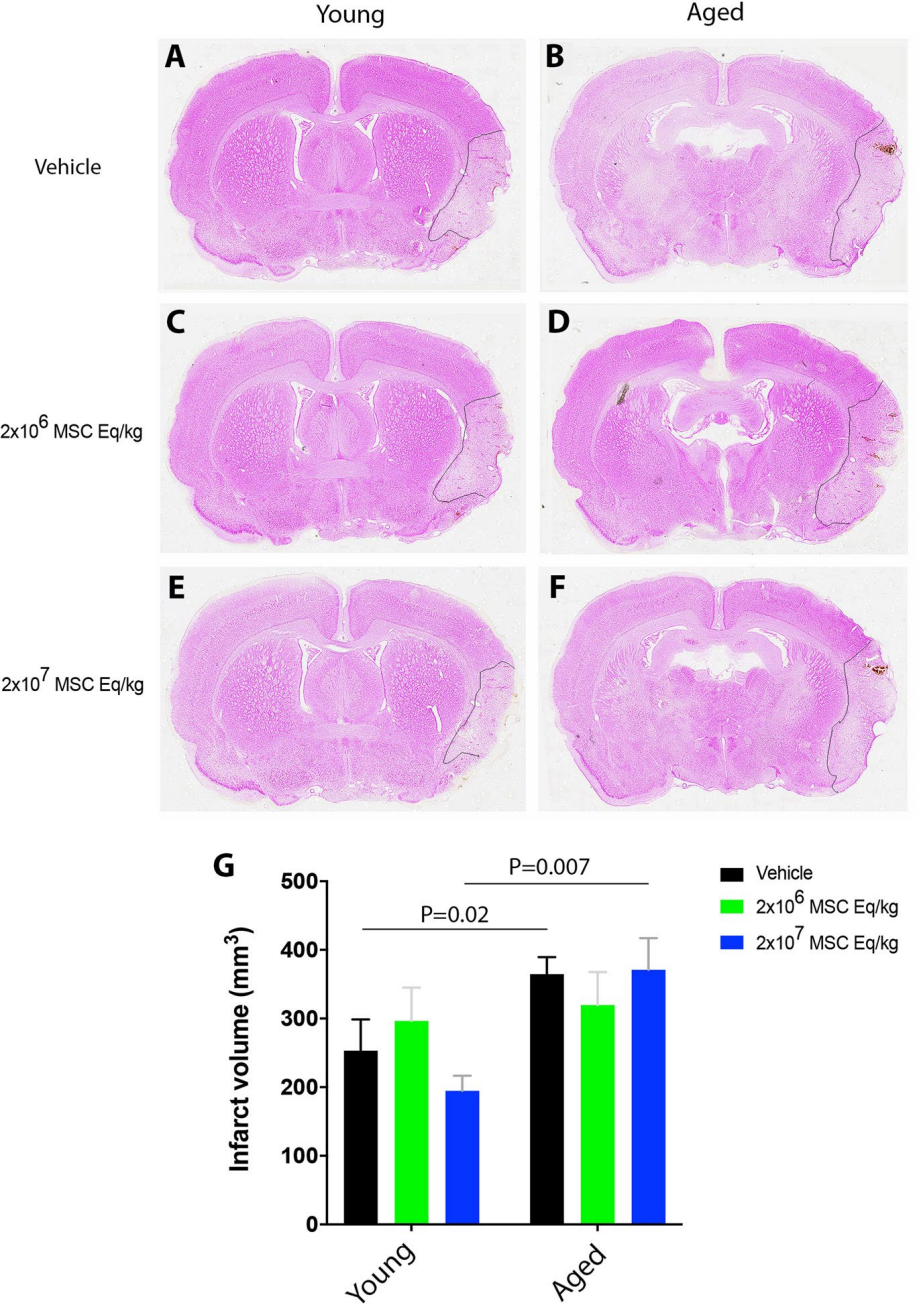
MSC-sEVs do not influence infarct volume in young or aged rats. Infarct volume assessed by methyl green/pyronine Y staining in **A**, **C**,** E** young and **B**, **D**,** F** aged rats exposed to permanent distal MCAO, which received vehicle or sEVs (2 × 10^6^ or 2 × 10^7^ MSC equivalents/kg) at 1, 3, and 7 days post-stroke, followed by animal sacrifice after 28 days (*n* = 15 animals/group). Note that **G** infarct volume was significantly larger in aged than young control rats. Data are mean ± SD values. Scale bar, 1 mm

They check if the delayed sEV treatment influenced infarct volume in this model of permanent distal MCAO; brain sections were stained with methyl green/pyronine Y following animal sacrifice at 28 days post-stroke (see Fig. 1A). At this time-point, the infarcted brain tissue had largely been resorbed. Yet, brain infarcts were detectable as focal lesions of the lateral parietal cortex, and the adjacent cortex exhibited cortical thinning as a sign of brain atrophy (Fig. 3A–F). Infarct volume determined using the indirect measurement technique (i.e., by evaluating viable tissue in both hemispheres) was significantly larger in aged than young vehicle-treated rats (1.86 times; *p* = 0.04) (Fig. 3G). Infarct volume was not influenced by low-dose or high-dose sEVs in young or aged rats (Fig. 3G), which is in line with previous studies following delayed MSC-sEV treatment [4].

### sEVs at both doses decrease the accumulation of ED1^+^ macrophages in the periinfarct cortex of aged rats

**Fig. 4:**
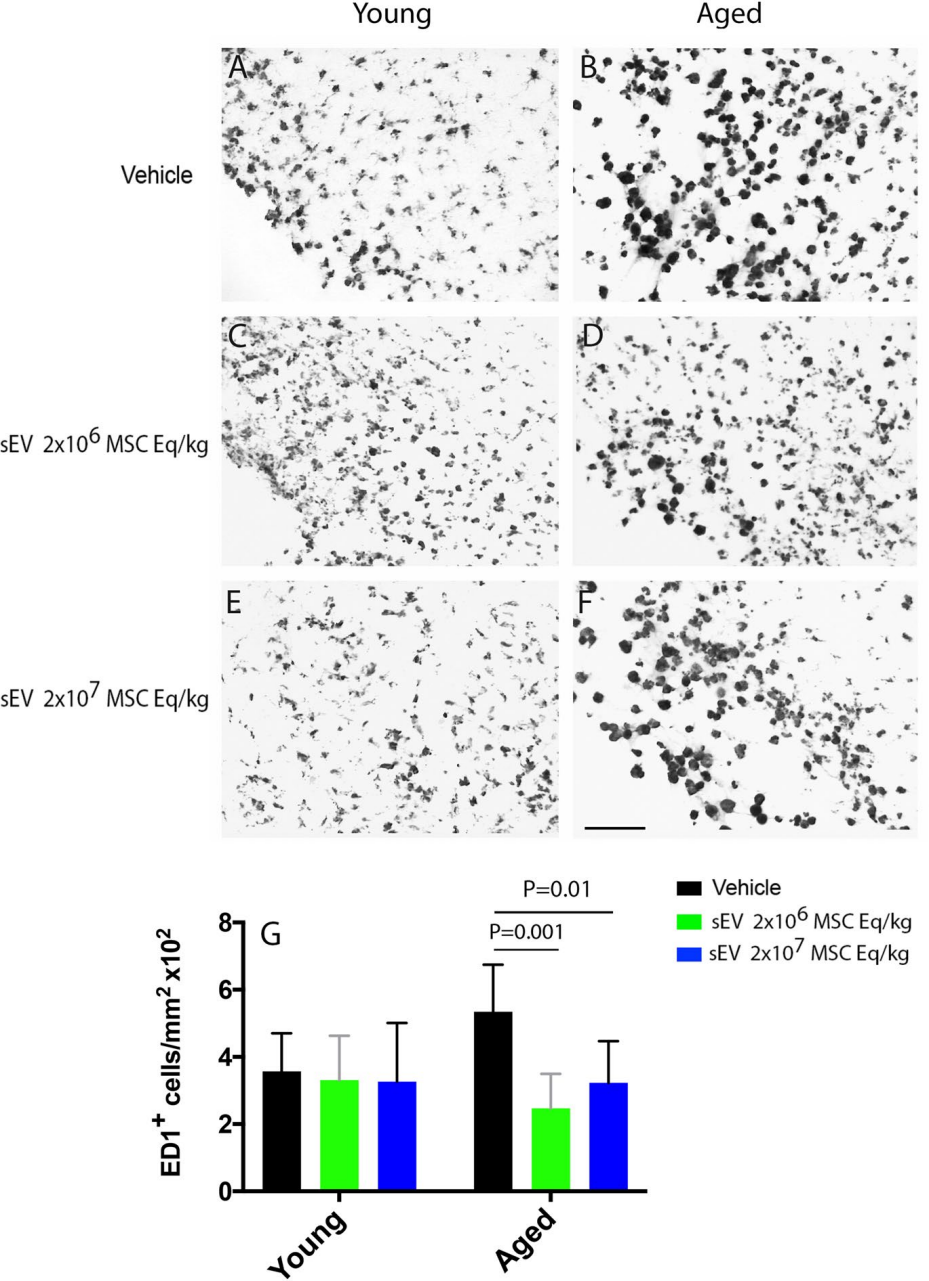
sEVs at both doses reduce periinfarct macrophage accumulation in aged rats. Number of ED1^+^ macrophages in the periinfarct cortex of **A**, **C**, **E** young and **B**, **D**,** F** aged MCAO rats assessed by immunohistochemistry. Rats received vehicle or sEVs (2 × 10^6^ or 2 × 10^7^ MSC equivalents/kg) at 1, 3, and 7 days post-stroke, followed by animal sacrifice after 28 days (*n* = 15 animals/group). Macrophages were identified using thresholds reflecting their appropriate size, which is ~ 50% larger than that of monocytes. Hence, monocytes were excluded from cell countings. Note G the exacerbated brain infiltrates of ED1^+^ macrophages in the brains of aged compared with young mice, which were markedly reduced by sEVs. sEVs did not influence ED1^+^ macrophage infiltrates in young mice. Data are mean ± SD values. Scale bar, 100 µm.

Ischemic stroke induces a proinflammatory milieu in the brain parenchyma, which impedes successful tissue remodeling and can be shifted to an immunotolerant proregenerative state by MSC-sEVs [6]. Indeed, MCAO induced robust brain inflammatory responses, indicated by the brain entry of ED1^+^ macrophages into the ischemic brain hemisphere after 28 days, which were discriminated from monocytes based on their characteristic size (Fig. 4A–F). The number of infiltrating ED1^+^ macrophages in the periinfarct cortex was significantly higher in aged than young rats (1.54 times; Fig. 4G). In aged rats, sEV treatment at both doses significantly reduced ED1^+^ brain macrophage infiltrates (*p* < 0.001 each for low-dose and high-dose sEVs; Fig. 4G).

### Low-dose sEVs reduce the accumulation of Iba1^+^ microglia in the periinfarct cortex of young rats

**Fig. 5:**
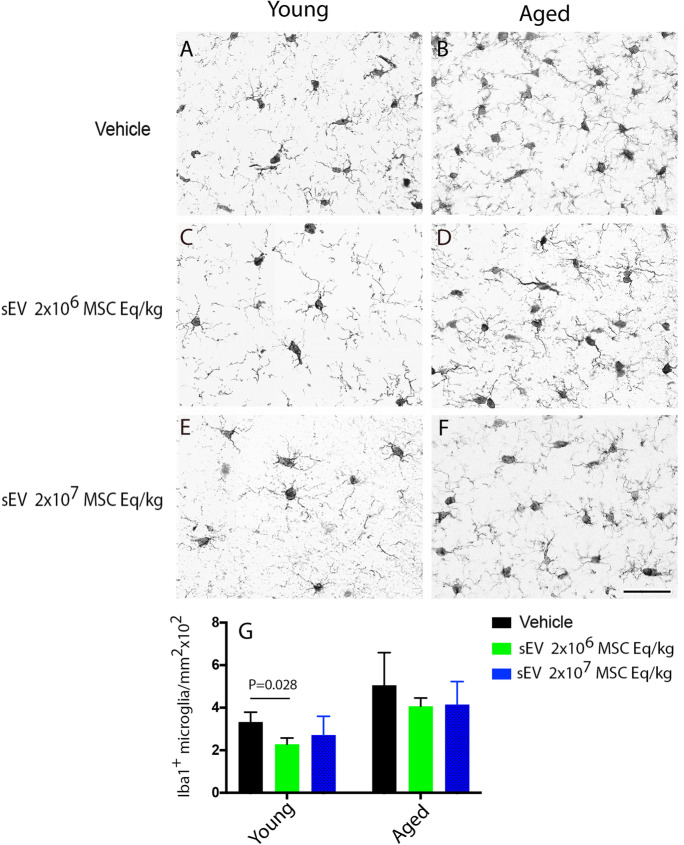
Low-dose sEVs reduce periinfarct microglia accumulation in young rats. Number of Iba1^+^ microglia in the periinfarct cortex of **A**, **C**, **E** young and **B**, **D**,** F** aged MCAO rats assessed by immunohistochemistry. Rats received vehicle or sEVs (2 × 10^6^ or 2 × 10^7^ MSC equivalents/kg) at 1, 3, and 7 days post-stroke, followed by animal sacrifice after 28 days (*n* = 15 animals/group). Note G the moderately elevated microglial recruitment in aged compared to young rats. Data are mean ± SD values. Scale bar, 50 µm

In line with macrophages, Iba1^+^ microglia, which are brain-resident myeloid cells responsible for first-line immune defense, abundantly accumulated in the periinfarct rim at 28 days post-stroke (Fig. 5A–F). Hence, we evaluated how sEV delivery influenced microglia abundance. Although the number of Iba1^+^ microglia in the periinfarct cortex was nominally higher in aged than young rats (1.51 times; Fig. 5G), this difference was statistically not significant. sEVs nominally reduced the number of Iba1^+^ microglia in young and aged rats (Fig. 5G). This effect was significant for low-dose sEVs in young rats (*p* = 0.028).

### sEVs at both doses promote angiogenesis in the periinfarct cortex of young and aged rats

**Fig. 6:**
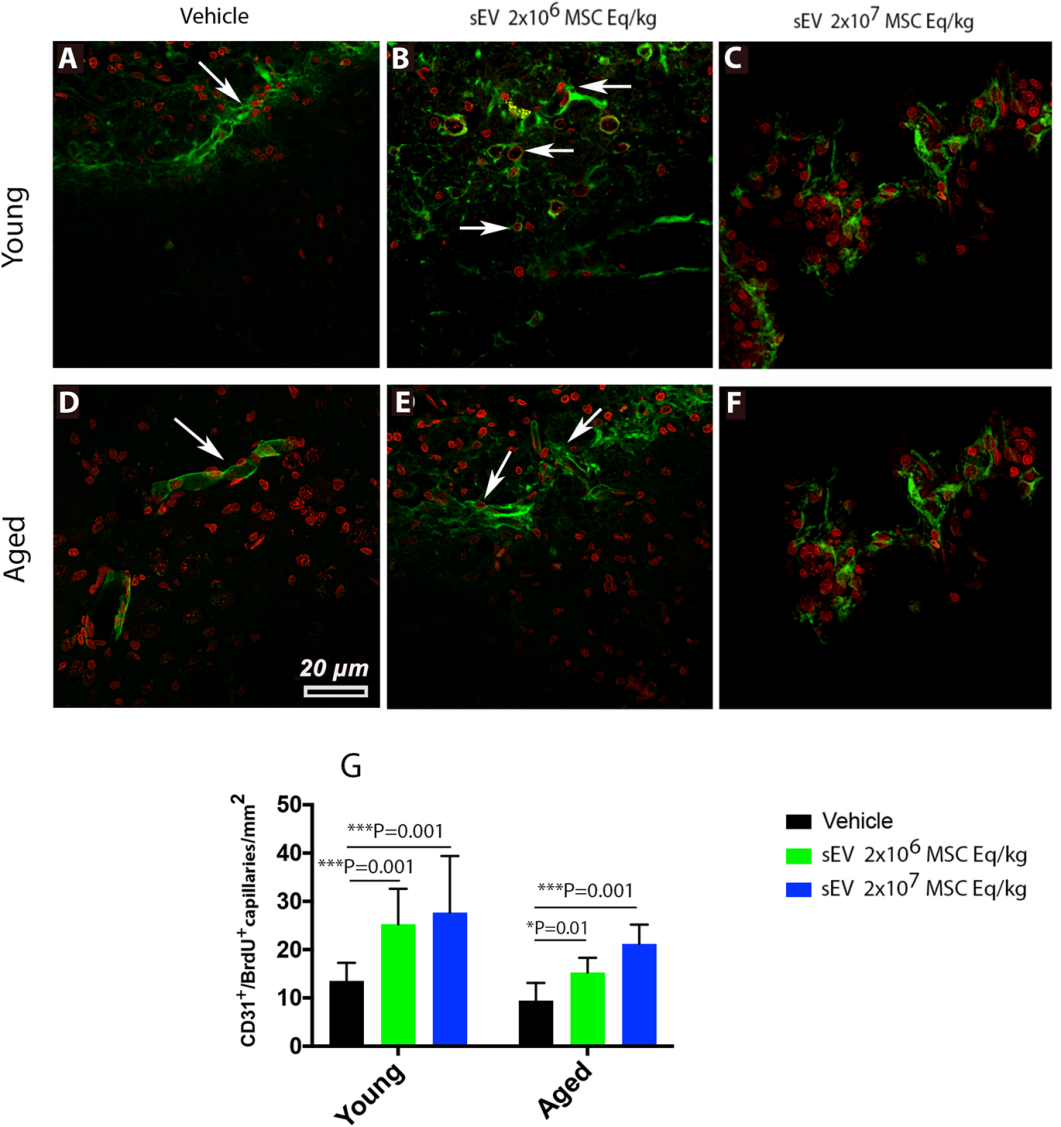
sEVs at both doses increase periinfarct angiogenesis in young and aged rats. Number of CD31^+^ brain microvessels (in green) in the periinfarct cortex of **A**, **B**, **C** young and **D**, **E**, **F** aged MCAO rats that were double labeled with the proliferation marker bromodeoxyuridine (BrdU; in red). Rats received vehicle or sEVs (2 × 10^6^ or 2 × 10^7^ MSC equivalents/kg) at 1, 3, and 7 days post-stroke (*n* = 15 animals/group). For the labeling of proliferating cells, BrdU (50 mg/kg body weight) was intraperitoneally administered daily from 8 to 18 days, followed by animal sacrifice after 28 days. Note G the dose-dependent stimulation of angiogenesis by sEVs in young and aged rats. Arrows depicting CD31^+^/BrdU^+^ double labeled cells. Data are mean ± SD values. Scale bar, 20 µm.

Considering that MSC-sEVs reversed the proinflammatory milieu in the brains of aged rats post-MCAO, we hypothesized that this shift in immune balance favored post-ischemic brain tissue remodeling. Brain hypoxia and ischemia are potent triggers of cerebral angiogenesis, which closely accompanies successful brain remodeling [28]. Indeed, we previously showed that MSC-sEVs promote post-ischemic angiogenesis in young mice exposed to proximal MCAO [4]. To evaluate the effects of sEVs on post-ischemic angiogenesis, we examined the number of CD31^+^/BrdU^+^ (that is, proliferating) microvessels in the periinfarct cortex of rats exposed to distal MCAO. In the unlesioned hemisphere, microvascular proliferation was rarely detectable by CD31/BrdU immunofluorescence in young and aged rats (not shown). In the periinfarct cortex, on the contrary, CD31^+^/BrdU^+^ microvessels were frequently noted (Fig. 6A–F). The number of CD31^+^/BrdU^+^ microvessels was similar in the periinfarct cortex of young and aged vehicle-treated rats (Fig. 6G). sEVs at both doses significantly increased the number of CD31^+^/BrdU^+^ microvessels in the periinfarct cortex of young (by 1.8 and 2.0 times for low-dose and high-dose sEVs, *p* < 0.001 each) and aged (by 1.6 and 2.2 times for low-dose and high-dose sEVs; *p* = 0.013 and *p* < 0.001, respectively) rats (Fig. 6G).

### Low-dose sEVs stimulates neurogenesis in the SVZ of young and aged rats

**Fig. 7:**
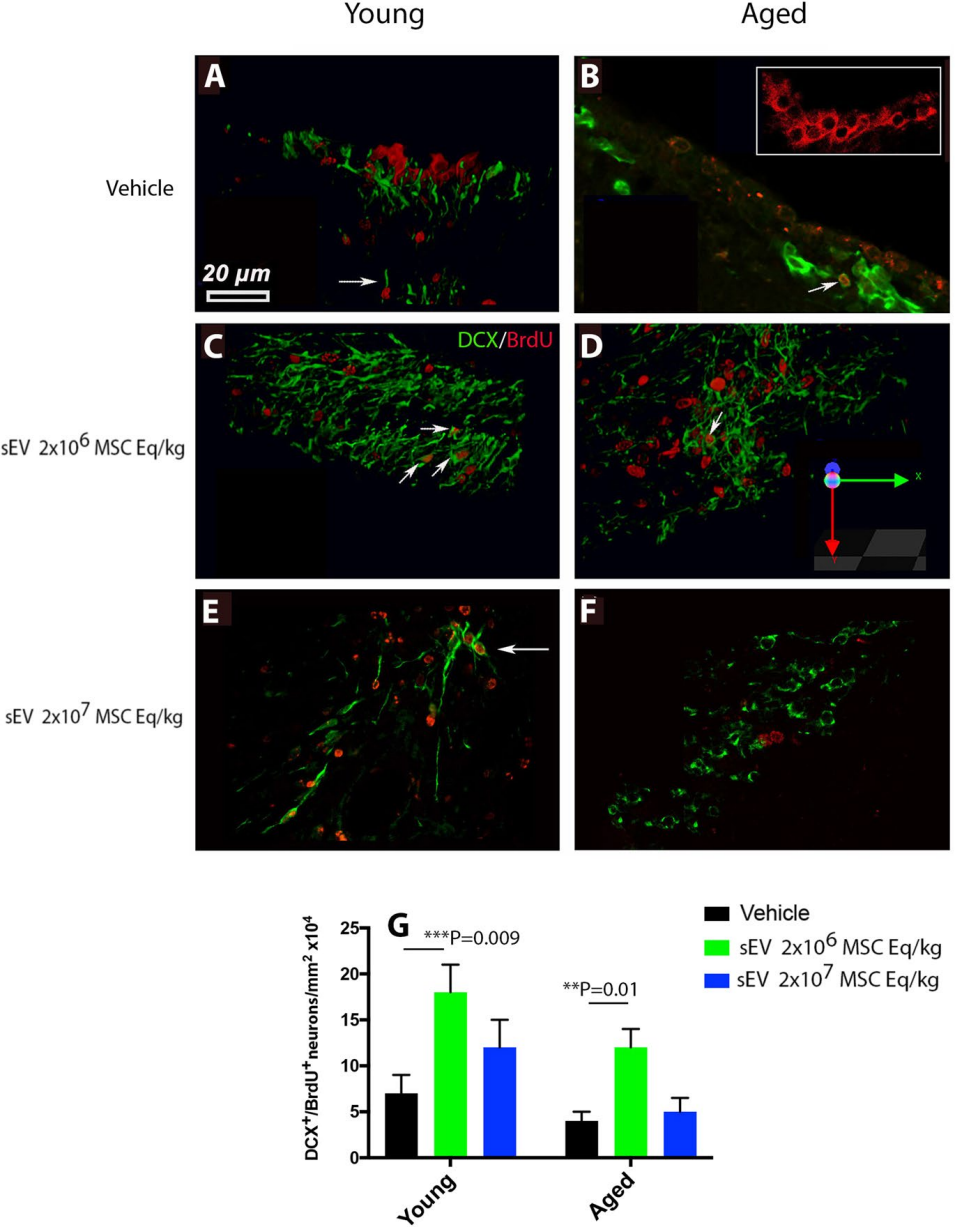
Low-dose sEVs stimulates post-stroke neurogenesis adjacent to the subventricular zone of young and aged rats. Number of newborn neurons adjacent to the subventricular zone (SVZ) of **A**, **C**, **E** young and **B**, **D**,** F** aged MCAO rats expressing the immature neuronal marker doublecortin (DCX; in green) that were double labeled by the proliferation marker BrdU (in red). Rats received vehicle or sEVs (2 × 10^6^ or 2 × 10^7^ MSC equivalents/kg) at 1, 3, and 7 days post-stroke (*n* = 15 animals/group). For cell proliferation analysis, BrdU (50 mg/kg body weight) was intraperitoneally administered daily from 8 to 18 days, followed by animal sacrifice after 28 days. Note G that low-dose sEVs increased endogenous neurogenesis in young and aged rats, whereas high-dose sEVs did not have any significant effect. Note that most DCX^+^ cells did not colocalize with BrdU^+^ nuclei in the SVZ of vehicle-treated rats (**A**, **B**; arrows). Instead, the BrdU^+^ nuclei were distributed mainly in a “pinwheel” configuration adjacent to the periventricular epithelium (**B**, inset). Arrows in (**C**–**E** depicting DCX^+^/BrdU^+^ cells. Data are mean ± SD values. Scale bar, 20 µm

Brain ischemia stimulates endogenous neurogenesis in the ipsilateral SVZ [29], which was previously shown to be increased by MSC-sEVs in young mice after proximal MCAO [4]. To clarify if endogenous neurogenesis was also elevated by sEVs in the distal rat MCAO model, we evaluated the number of BrdU^+^ newborn neurons adjacent to the SVZ that expressed the immature neuronal marker doublecortin (DCX). The number of DCX^+^/BrdU^+^ newborn neurons was higher in young than aged rats (Fig. 7A–G). Low-dose sEVs increased the number of DCX^+^/BrdU^+^ neurons in young (2.6 times, *p* = 0.009) and aged (2.6 times, *p* = 0.010) rats (Fig. 7G). Interestingly, high-dose sEVs did not influence the number of DCX^+^/BrdU^+^ neurons in either age group (Fig. 7G). In vehicle-treated rats, most DCX^+^ cells adjacent to the SVZ did not colocalize with BrdU^+^ nuclei (Fig. 7A, B; arrows). Instead, the BrdU^+^ nuclei were distributed mainly in a “pinwheel” configuration adjacent to the periventricular epithelium (Fig. 7B, inset).

## Discussion

In a head-to-head comparison of young and aged rats exposed to permanent distal MCAO, we show that MSC-derived sEVs promote neurological recovery and brain remodeling in aged rats, when administered in the post-acute stroke phase starting 24 h post-stroke. Although aged rats exhibited more severe motor-coordination deficits evaluated by rotating pole and cylinder tests and larger brain infarcts than young rats, MSC-sEVs at low (three times 2 × 10^6^ MSC equivalents/kg at 24 h, 3 and 7 days) and high (three times 2 × 10^7^ MSC equivalents/kg) dosage very effectively improved motor-coordination deficits starting at 7 days post-stroke (i.e., 6 days post-treatment onset) in aged rats. Notably, the effect of MSC-sEVs on motor-coordination recovery was more pronounced in aged than young rats, and it persisted across the observation period of 28 days. Infarct volume after animal sacrifice was not influenced by MSC-sEVs in either young or aged rats. The absence of infarct volume changes is in line with previous observations of our group following delayed MSC-sEV delivery starting at 24 h post-MCAO in young mice [4]. In this earlier study, we compared the efficacy of MSC-sEVs and their parental MSCs in a transient proximal MCAO model using a test battery consisting of rotarod, tight rope, and corner turn tests and demonstrated that MSC-sEVs and MSCs equally effectively promote post-stroke motor-coordination recovery [4]. The treatment timing in this earlier study was identical to the present study, in which we now confirmed the efficacy of sEVs in a distal MCAO model in young and aged rats.

Meanwhile several studies confirmed recovery-promoting effects of MSC-sEVs in ischemic stroke models in young mice and rats [1, 2, 4, 10–14, 16–18, 30, 31]. These studies employed permanent [14, 30, 31] and transient [1, 2, 4, 10, 13, 16–18] proximal MCAO models, which were induced using an intraluminal monofilament technique [1, 2, 4, 10–13, 16–18, 30, 31] or by local endothelin administration [14]. To the best of our knowledge, effects of MSC-sEVs on neurological recovery had not been shown in aged rodents and in a distal MCAO model. Distal MCAO differs from proximal MCAO that the cortex but not striatum is injured by the stroke.

So far, only one study examined the effects of intravenous MSC-sEV delivery on post-stroke motor-coordination recovery in middle-aged (12-month-old) mice [32]. This study used a thromboembolic stroke model. Perhaps due to differences in experimental protocols or MSC properties — MSCs were raised from embryonic stem cells — no recovery-promoting effects were noted [32]. We have previously shown that the efficacy of MSC-sEVs critically depends on the MSC source, cell culturing conditions, and sEV preparations [12, 16]. Only by thorough characterization of both the MSCs and their sEVs, the quality of MSC-sEV preparations can be assured. In accordance with our previous studies [12, 16], we performed an in depth analysis of sEV preparations (Suppl. Figure 1, Suppl. Tables 1 and 2) according to current International Society of Extracellular Vesicle (ISEV) guidelines [20]. The same MSC source (41.5) as in our previous studies [12, 16] was used. MSCs were cultured, and sEV preparations were purified identically as previously reported [12, 16].

Ischemic stroke is associated with a proinflammatory brain microenvironment, which impedes brain remodeling and neurological recovery after stroke [16]. In the present study, ED1^+^ macrophage infiltrates were markedly increased in the periinfarct cortex of aged compared with young rats, indicating that post-ischemic neuroinflammation is exacerbated upon ageing. Importantly, MSC-sEVs reduced ED1^+^ macrophage infiltrates in aged rats. Our study expands previous observations of our group in young mice exposed to proximal (i.e., intraluminal) MCAO, showing that MSC-sEVs reduce brain infiltrates of polymorphonuclear neutrophils (PMNs), monocytes/macrophages, and lymphocytes in the periinfarct brain tissue [16]. In these earlier studies, PMN depletion using anti-Ly6G antibody delivery decreased brain monocyte/macrophage and lymphocyte infiltrates in the periinfarct tissue and mimicked the effects of MSC-sEVs on neurological deficits and ischemic brain injury [16]. Interestingly, MSC-sEVs failed to reduce neurological deficits and ischemic injury in PMN-depleted young mice [16]. Based on these observations we concluded that brain-infiltrating leukocytes play a central role in MSC-sEV-induced neuroprotection [16]. We hypothesized that MSC-sEVs exert their action by shifting the immune balance from a proinflammatory to an immunotolerant state [6]. By showing that brain macrophage infiltrates in aged rats are reduced by MSC-sEVs, we now revealed a powerful mechanism via which MSC-sEVs promote neurological recovery in aged rats.

In young, otherwise healthy rats and mice exposed to proximal MCAO, we and others have previously shown that MSC-sEVs promote post-ischemic brain parenchymal remodeling by stimulating periinfarct angiogenesis, neurite and synapse remodeling, and endogenous neurogenesis in the SVZ (Doeppner et al., 2015; Gregorius et al., 2021; Otero-Ortega et al., 2017; Xin et al., 2017; Xin et al., 2013). Angiogenesis, neuroplasticity, and neurogenesis are age-dependent processes [33] that are tightly linked post-stroke [28]. We therefore asked if the effects of MSC-sEVs on periinfarct angiogenesis and SVZ neurogenesis were attenuated in aged rats. Contrary to this assumption, MSC-sEVs at both doses potently increased post-ischemic angiogenesis in the periinfarct cortex in young and aged rats, whereas low-dose MSC-sEVs, but not high-dose MSC-sEVs increased neurogenesis in the SVZ. In vitro, sEVs isolated from rat cerebral microvascular endothelial cells have previously been shown to be internalized by axons cultivated from rat pups, in which they were found to promote axonal growth in an argonaute-2-dependent way [34].

In this study, only low-dose sEVs, but not high-dose sEVs increased post-ischemic neurogenesis in the SVZ. Our observations differ from previous studies after proximal MCAO in rats and mice, where robust endogenous neurogenesis has been reported following MSC-sEV delivery [2, 4]. In response to proximal MCAO, neural precursor cells of the SVZ proliferate and migrate in direction to the stroke lesion [35, 36]. By secreting growth factors, these neural precursor cells are thought to contribute to post-ischemic neurological recovery [28]. In contrast to proximal MCAO, which severely damages the striatum immediately adjacent to the SVZ, distal MCAO produces purely cortical brain infarcts at distance to the SVZ. The different stroke topography might explain why the stimulation of endogenous neurogenesis was less robust after distal MCAO than proximal MCAO.

Our observation of the efficacy of MSC-sEVs in aged rats exposed to stroke complements a recent study in Rhesus monkeys receiving cold lesions of the primary M1 motor cortex, in which MSC-sEV delivery enhanced fine motor movement recovery [37, 38]. In this previous study, MSC-sEVs were intravenously administered at 24 h and 14 days post-cold injury. Grasp patterns of the contralesional paretic hand were examined for 12 weeks in a food retrieval task. Compared to vehicle-treated monkeys, grasp patterns of sEV-treated monkeys returned to pre-operative levels during the first 3 to 5 weeks post-stroke [38]. Supporting an immunomodulatory effect of MSC-sEVs, microglial accumulation and activation in the periinfarct cortex were reduced by MSC-sEVs [37].

## Conclusion

This study provides evidence that MSC-sEV delivery efficiently promotes neurological recovery and periinfarct brain remodeling after permanent distal MCAO in aged rats. This effect was attributed to the attenuation of brain macrophage infiltrates, which were markedly increased in aged compared with young rats. This study encourages further proof-of-concept studies evaluating MSC-sEV efficacy in clinic-relevant stroke settings.

## Supplementary Information

Below is the link to the electronic supplementary material.

Supplementary file1 (DOCX 216 KB)

## Data Availability

Data will be made available to qualified researchers upon request.
