# Supplementary material for: Mesenchymal stromal cell-derived small extracellular vesicles promote neurological recovery and brain remodeling after distal middle cerebral artery occlusion in aged rats
Source: GeroScience. 2021 Nov 10;44(1):293–310. doi: 10.1007/s11357-021-00483-2 (PMC8811093; doi:10.1007/s11357-021-00483-2)
Supplement: Supplementary file 1 — Supplementary file1 (DOCX 216 KB) [file 11357_2021_483_MOESM1_ESM.docx]

**SUPPLEMENTAL MATERIAL**

**Mesenchymal stromal cell-derived small extracellular vesicles promote neurological recovery and brain remodeling after distal middle cerebral artery occlusion in aged rats**

Danut Dumbrava^1^*, Roxana Surugiu^1^*, Verena Börger^2^, Mihai Ruscu^1^, Tobias Tertel^2^, Bernd Giebel^2^, Dirk M. Hermann^1,3^*^#^, Aurel Popa-Wagner^1,3,4^*^#^

^1^Experimental Research Center in Normal and Pathological Aging (ARES), University of Medicine and Pharmacy, Craiova, Romania; ^2^Institute for Transfusion Medicine and ^3^Department of Neurology, University Hospital Essen, University of Duisburg-Essen, Essen, Germany; ^4^Griffith University Menzies Health Institute of Queensland, Gold Coast Campus, Gold Coast Campus, QLD 4222, Australia; *equally contributing first/senior authors, ^#^corresponding authors

Number of supplementary figures: 1

Number of supplementary tables: 3

**Correspondence to:**

aurel.popa-wagner@geriatrics-healthyageing.com or

dirk.hermann@uk-essen.de

**Supplemental Figures:**


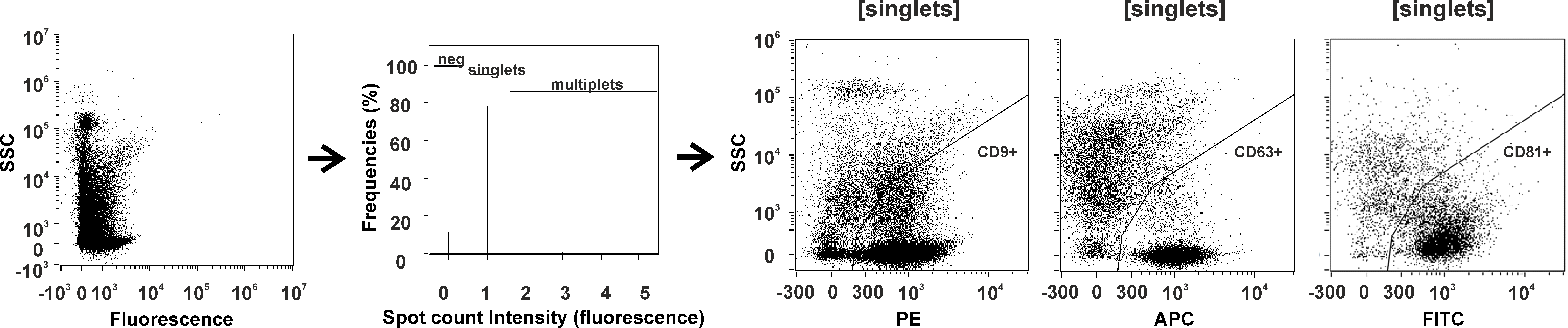


**Supplementary Figure 1**: **Gating strategy for measurement of sEVs by ImageStreamX flow cytometry.** From all recorded signals (1^st^ plot from left), signals not showing spot counts or signal multiplets were excluded (2^nd^ plot from left). In the three representative plots on the right, side scatter (SSC) intensities of single objects are plotted against the fluorescence intensities of CD9^+^ (labeled with PE), CD63^+^ (labeled with APC) or CD81^+^ (labeled with FITC) objects.

**Supplementary Tables:**

**Supplementary Table 1: Biophysical characteristics of MSC-sEV preparations.**

|  | Particle concentration [particles/ ml] | Particle size [nm] | Protein concentration [µg/ µl] | Purity [particles/ mg protein] |
| --- | --- | --- | --- | --- |
| Preparation A | 3.8x10^11^ | 125.6 | 7.84 | 4.8x10^10^ |
| Preparation B | 1.5x10^11^ | 119.2 | 5.48 | 2.7x10^10^ |

Particle size is given as mean ± standard deviation (SD) value. Preparation A was administered to young rats, and preparation B to aged rats.

**Supplementary Table 2: Flow cytometric characterization of MSC-derived sEV preparations**.

| EV marker | CD9^+^ objects [/ ml] | CD63^+^ objects [/ ml] | CD81^+^ objects [/ ml] |
| --- | --- | --- | --- |
| Preparation A | 1.62 ± 0.57 x 10^8^ | 2.90 ± 0.56 x 10^6^ | 1.52 ± 0.85 x 10^8^ |
| Preparation B | 1.46 ± 0.23 x 10^9^ | 1.55 ± 1.63 x 10^8^ | 7.23 ± 0.56 x 10^6^ |

Number of objects per milliliter evaluated by AMNIS ImageStreamX analysis. Data are means ± SD values. Preparation A was administered to young rats, and preparation B to aged rats.

**Supplementary Table 3: Antibodies used for flow cytometry.**

| Antigen | Conjugate | Host/isotype | Clone | Supplier |
| --- | --- | --- | --- | --- |
| Human CD9 | PE | Mouse, IgG1 | MEM-61 | Exbio |
| Human CD63 | APC | Mouse, IgG1 | MEM-259 | Exbio |
| Human CD81 | FITC | Mouse, IgG2a | JS64 | Beckman-Coulter |
